# Black silicon significantly enhances phosphorus diffusion gettering

**DOI:** 10.1038/s41598-018-20494-y

**Published:** 2018-01-31

**Authors:** Toni P. Pasanen, Hannu S. Laine, Ville Vähänissi, Jonas Schön, Hele Savin

**Affiliations:** 1 Aalto University, Department of Electronics and Nanoengineering, Espoo, 02150 Finland; 2 Fraunhofer Institute for Solar Energy Systems, Freiburg, 79110 Germany; 3 University of Freiburg, Department of Sustainable Systems Engineering, Freiburg, 79110 Germany

## Abstract

Black silicon (b-Si) is currently being adopted by several fields of technology, and its potential has already been demonstrated in various applications. We show here that the increased surface area of b-Si, which has generally been considered as a drawback e.g. in applications that require efficient surface passivation, can be used as an advantage: it enhances gettering of deleterious metal impurities. We demonstrate experimentally that interstitial iron concentration in intentionally contaminated silicon wafers reduces from 1.7 × 10^13^ cm^−3^ to less than 10^10^ cm^−3^ via b-Si gettering coupled with phosphorus diffusion from a POCl_3_ source. Simultaneously, the minority carrier lifetime increases from less than 2 μs of a contaminated wafer to more than 1.5 ms. A series of different low temperature anneals suggests segregation into the phosphorus-doped layer to be the main gettering mechanism, a notion which paves the way of adopting these results into predictive process simulators. This conclusion is supported by simulations which show that the b-Si needles are entirely heavily-doped with phosphorus after a typical POCl_3_ diffusion process, promoting iron segregation. Potential benefits of enhanced gettering by b-Si include the possibility to use lower quality silicon in high-efficiency photovoltaic devices.

## Introduction

The special properties of black silicon (nanostructured silicon, b-Si), such as large surface area, negligible reflectance and superhydrophobicity, provide interesting opportunities in various applications, including biological and chemical sensors^1^, self-cleaning surfaces^2^, and photodiodes^3^. Currently b-Si is of particular interest within the photovoltaics industry^4,5^ due to its high compatibility with recently introduced diamond wire-sawn multicrystalline silicon wafers^6^.

Although several interesting properties of b-Si have been extensively studied in recent years, many potential advantages have yet remained undiscovered. One advantage worth studying is the potentially enhanced gettering of metal impurities which are deleterious in several applications, including transistors and photovoltaic devices^7^. In gettering, the impurities are typically relocated to less critical sites within the device. This is often realised via impurity segregation to a heavily phosphorus-doped region^8–11^, typically referred as phosphorus diffusion gettering (PDG). Since the b-Si nanostructures are typically more heavily doped than planar wafers after dopant diffusion^12–14^, b-Si is likely to enhance PDG via increased segregation.

In addition to enhanced segregation, the large surface area or possible structural defects induced by b-Si fabrication^15–17^ may provide additional gettering sites by introducing nucleation sites for metal precipitates. An idea of enhanced gettering via increased surface area in silicon solar cells was proposed by Dimassi *et al*.^18^. They fabricated sacrificial porous silicon (PS) layers on Si wafers and demonstrated that the harmful impurities were gettered from the bulk material to the porous layer during thermal annealing due to the high surface reactivity of PS. Similarly, mechanical^19^ or saw damage^20,21^ has been used as effective gettering sites. Thus, b-Si could promote the removal of impurities via near-surface precipitation, in addition to enhanced segregation.

We perform here a benchmark study to quantitatively compare the gettering performance of b-Si and planar reference wafers for iron, the most deleterious metal impurity in p-type silicon^7,22^. We intentionally contaminate IC-grade CZ-Si wafers with iron at specific concentrations and manufacture b-Si on these substrates. The wafers are subjected to PDG processes with varying temperature profiles, and the resulting gettering efficiencies are characterised by minority charge carrier lifetime measurements. Finally, we draw guidelines toward implementing these findings into predictive process simulators^23,24^ via discussing the dominant gettering mechanisms and compare the experimental findings with simulations.

## Methods

**Figure 1:**
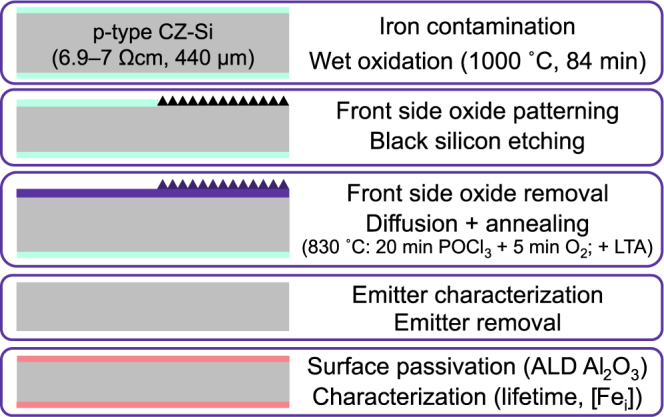
Experimental procedure.

**Figure 2:**
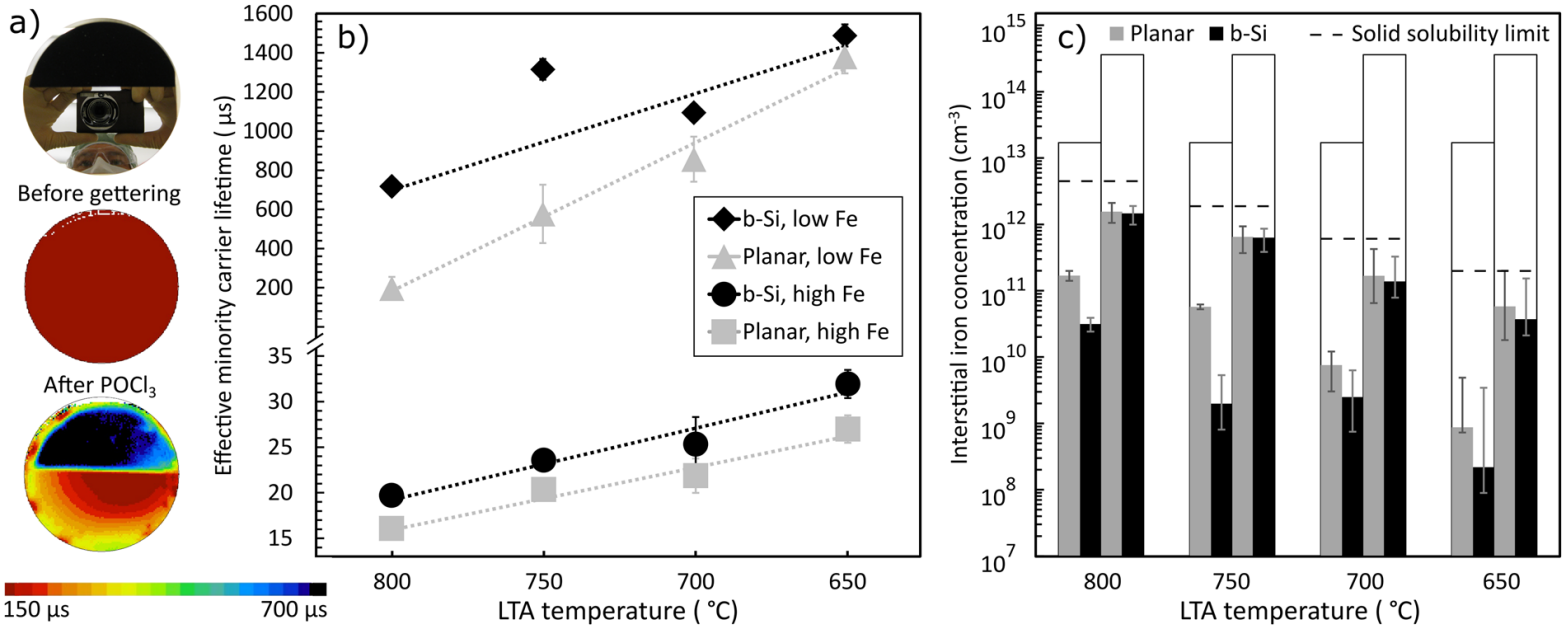
(**a**) A photograph showing b-Si etched on half of each wafer and μ-PCD lifetime maps of a contaminated wafer without gettering and a selected gettered wafer (800 °C LTA, low Fe). (**b**) Effective minority carrier lifetimes of the passivated wafers LTA treated at different temperatures. Note the break in the y-axis. The reported values have been averaged from μ-PCD maps measured before Fe-B pair dissociation. The dashed lines act as a guide for eye. The error bars have been determined from the range of variation in lifetime within the μ-PCD maps. (**c**) Interstitial iron concentration of the gettered b-Si and planar wafer halves determined from injection-level dependent QSSPC measurements. The left-hand bars represent the low Fe wafers and the right-hand bars the high Fe samples as indicated by the white bars which denote the interstitial iron concentration of the ungettered reference wafers. The dashed lines indicate the solid solubility limit of iron at each temperature according to^42^. Note the logarithmic y-axis. The error bars have been determined assuming a 5% uncertainty in the QSSPC measured lifetime.

The experimental procedure is outlined in Fig. 1. First, a controlled contamination procedure^10^ was carried resulting in two different initial total iron levels typically found in high-performance multicrystalline silicon^22^: 1.7 × 10^13^ cm^−3^ (low) and 3.6 × 10^14^ cm^−3^ (high). After wet oxidation, b-Si was fabricated on one half of each wafer by cryogenic deep reactive ion etching (RIE) using process parameters described in^25^. As a result, every wafer had both b-Si and planar halves (Fig. 2a) to make the comparison between the two surfaces reliable and straightforward. A phosphorus-doped layer was formed in the front side of the wafers by POCl_3_ diffusion. Subsequently, temperature was ramped down to 600–800 °C with a 4 °C/min rate to a low temperature anneal (LTA) step for 2 h at 800 °C, 3.5 h at 750 °C, 5.5 h at 700 °C or 8 h at 650 °C. The annealing times were chosen based on simulations to reach steady state iron concentration throughout the whole wafer^26^.

In order to determine the bulk minority charge carrier recombination lifetime – a proxy for cell efficiency potential^27^ – the heavily-doped layers were removed by wet etching. A 2-minute dip in a CH_3_COOH:HF:HNO_3_ solution etched approximately 5 µm from both surfaces of a planar reference wafer. The wafer surfaces were subsequently passivated with atomic-layer-deposited (ALD) aluminum oxide (Al_2_O_3_) using a process described in^14^. To determine the bulk interstitial iron concentration, lifetime was measured before and after Fe-B pair dissociation with both microwave-detected photoconductance decay (μ-PCD) and quasi-steady-state photoconductance (QSSPC) techniques. The iron concentration was determined from the measured QSSPC data per an extended model developed by Macdonald *et al*.^28^ at an injection level around 10^16^ cm^−3^.

Finally, simulations were carrier out to elucidate the underlying physical processes operating in the experiment. Phosphorus diffusion was simulated using Sentaurus Process by applying the model and parameters from^29^. In analogy to^13^, the phosphorus concentration in the phosphosilicate glass was fitted to the electrochemical capacitance-voltage (ECV) profile measured at planar wafers, and b-Si was treated as a triangular 2D structure in the simulations. The phosphorus profile in b-Si was averaged over a single needle in lateral direction to obtain a 1D profile comparable to the planar case. The resulting gettering efficiency was simulated using an iron segregation model involving iron point defects reacting with doubly-charged vacancies and pairing with positively charged phosphorus originally suggested by Haarahiltunen *et al*.^26^ with a parametrization revised by Talvitie *et al*.^9^.

## Results and Discussion

Figure 2a shows μ-PCD lifetime maps of a low Fe contamination level wafer without gettering and a corresponding wafer after POCl_3_ diffusion and a LTA at 800 °C for 2 h. The carrier lifetime significantly improves by the presence of b-Si. The lifetime in the b-Si half (720 μs) is more than two orders of magnitude higher than in the ungettered wafer (1.9 μs) and more than three times that of the gettered planar half (200 μs), implying a significantly higher cell efficiency potential for the b-Si wafers^27^. Since both halves have been passivated simultaneously, the lifetime difference is a direct result of differences in the wafer bulk, indicating more efficient gettering in b-Si. Possible slight surface roughness on the b-Si area after Si etching would be visible only as a reduced effective lifetime in the b-Si half, which indicates that the difference in bulk lifetime may be even higher.

Figure 2b presents the effective minority carrier lifetime averaged from μ-PCD maps for all the samples gettered with various LTA sequences. Regardless of the LTA conditions, b-Si results in a higher lifetime than a planar surface in both low and high Fe samples. As hypothesised, the increased carrier lifetime in the b-Si halves is indeed due to a reduced amount of iron in the bulk (Fig. 2c) caused by enhanced gettering. For instance, the lifetime map shown in Fig. 2a corresponds to final bulk iron concentrations of 3 × 10^10^ cm^−3^ and 2 × 10^11^ cm^−3^ in the b-Si and planar wafer halves, respectively.

The segregation efficiency improves with decreasing temperature due to a larger iron solubility difference between the heavily phosphorus-doped region and the wafer bulk^30^. This phenomenon is clearly visible in Fig. 2b and c as an increase in lifetime and decrease in iron concentration with reducing LTA temperature. Within the high Fe samples, carrier lifetimes in b-Si and planar wafer halves increase with equal rate within the measurement accuracy. In the low Fe samples instead, lifetime in the planar reference approaches b-Si with decreasing LTA temperature. Lifetime limited by surfaces (~1.5 ms) with a reasonable surface recombination velocity of 15 cm/s^31^ agrees with the highest measured value and is more than one order of magnitude lower than iron-limited lifetime with 10^9^ cm^−3^ impurity concentration^32^. Thus, the diminishing difference between b-Si and planar lifetimes with decreasing LTA temperature is due to surface recombination dominating the effective lifetime despite the efficient ALD Al_2_O_3_ passivation. Similar behavior is not observed in the iron concentration values since the effect of surfaces cancels out in its extraction. However, the flash used to dissociate Fe-B pairs may improve the efficiency of ALD Al_2_O_3_ surface passivation^33^, which would slightly overestimate the extracted iron concentration in the low Fe samples^28^. Nevertheless, this does not affect the comparison between b-Si and planar samples.

**Figure 3:**
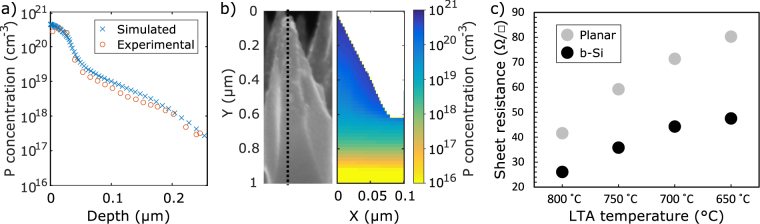
(**a**) Experimental and simulated phosphorus profiles of a planar sample POCl_3_-diffused at 830 °C for 20 min with no low temperature anneal. The experimental ECV data is from^14^. Note the logarithmic y-axis. (**b**) A SEM image of a single b-Si needle and the corresponding simulated phosphorus profile. (**c**) Experimental sheet resistance values for the b-Si and planar samples after various LTA treatments.

An explanation for the enhanced gettering is likely to be the high phosphorus concentration in the b-Si nanostructure. To further investigate this hypothesis, phosphorus diffusion into b-Si was simulated since the experimental determination of doping profile in the nanoscopic b-Si needles is challenging. Figure 3a verifies the used models by showing a good fit between the experimental and simulated profiles of electrically active phosphorus in a planar wafer diffused at 830 °C for 20 minutes. A corresponding simulated 2D doping profile in b-Si is presented in Fig. 3b, which shows that the silicon needles are extremely heavily doped with phosphorus. Furthermore, the simulations show that the active phosphorus dose in b-Si is three to four times that in the planar wafers. Also the experimental sheet resistance values shown in Fig. 3c illustrate that the doping concentration in b-Si is always considerably higher than in the planar samples.

The sheet resistance varies with LTA temperature (Fig. 3c), due to two effects. First, at higher temperatures, the phosphorus diffusivity is higher, allowing more phosphorus to diffuse into the wafer. On the other hand, with reducing LTA temperature a larger portion of phosphorus precipitates and turns electrically inactive due to reduced phosphorus solid solubility. Nevertheless, although doping concentration is higher in the samples with higher LTA temperature, which would enhance segregation, the temperature dependence of iron segregation coefficient dominates the observed trend in interstitial iron concentration (Fig. 2c), resulting in more iron gettered with lower temperatures.

**Figure 4:**
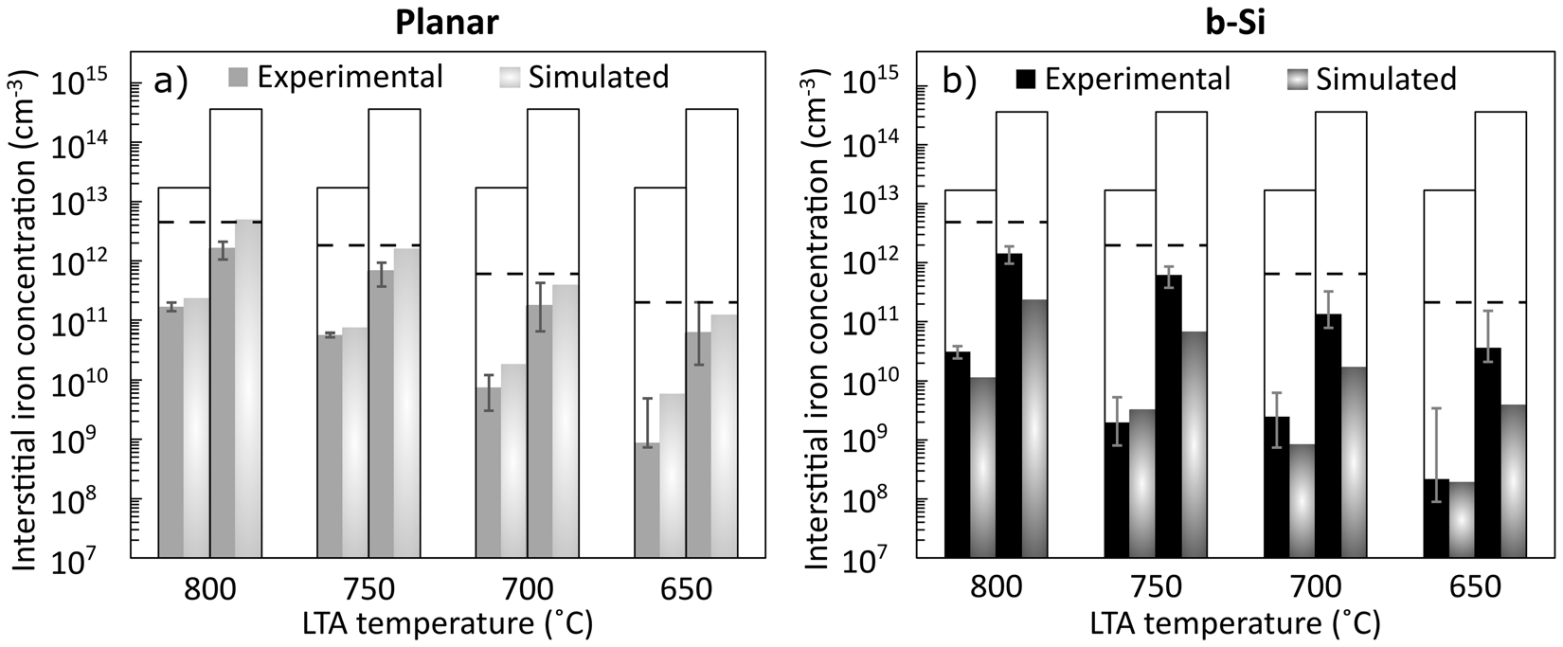
Comparison of experimental and simulated interstitial iron concentration in planar (**a**) and b-Si (**b**) samples. The left-hand bars represent the low Fe wafers and the right-hand bars the high Fe samples as indicated by the open bars which denote the interstitial iron concentration of the ungettered reference wafers. The dashed lines indicate the solid solubility limit of iron at each temperature according to^42^. Note the logarithmic y-axes. The error bars are determined assuming a 5% uncertainty in the QSSPC measurements.

The final iron concentrations are below the solid solubility limits at every temperature (Fig. 2c), which suggests segregation to be the dominant gettering mechanism. Similarly, the observation that the gettering efficiency is rather independent of initial iron concentration is also characteristic of segregation^7^. Furthermore, activation energies calculated from the low Fe data are 2.4–2.5 eV for both b-Si and planar samples, which agree well with the previously reported 2.5 eV activation energy for the segregation coefficient^9^. Figures 4a and b compare simulated bulk iron concentrations, with segregation gettering as the only active gettering mechanism^9,26^, to the experimental values in planar and b-Si samples, respectively. In all cases, the trends of the simulated and experimental results are consistent, further supporting our hypothesis that segregation is the main gettering mechanism. In the b-Si samples, however, the simulation predicts lower iron concentrations than are experimentally observed. Nevertheless, the differences can be attributed to challenges in accurate quantitative simulation of dopant diffusion and segregation in b-Si, and measuring iron accurately at concentrations below ~5 × 10^9^ cm^−3^^28^.

Among planar samples, also the quantitative results mainly agree with the experiments within the accuracy limits (Fig. 4a). However, the experiments systematically show slightly more efficient gettering than the simulation predicts, which could be explained by iron precipitation within the wafer bulk. This hypothesis is supported by the experimental iron concentrations of high Fe samples, where the difference between b-Si and planar halves is not as evident as in the low Fe samples (Fig. 2c). As the initial iron concentration in the high Fe samples is well above the iron solid solubility limit, iron starts to precipitate in the bulk during the LTA sequence^34^. As bulk precipitation is independent of wafer surfaces, the phenomenon reduces interstitial iron concentration in b-Si and planar samples to a similar degree. Nevertheless, iron segregation is likely dominant also in the high Fe samples since the final iron concentrations are below the solid solubility limits and the difference in gettering efficiency is visible in carrier lifetime (Fig. 2b).

No evidence was observed within this study for gettering enhancement by RIE-caused surface damage. On the other hand, earlier microscopic investigations of the specific b-Si fabrication process used here found little to no damage^35^. Some other b-Si fabrication methods, such as metal-assisted chemical etching^36,37^, introduce contaminants, and b-Si gettering could be efficiently used to remove the residual metals afterwards. The gettering efficiency may have been intensified by iron accumulation at the Al_2_O_3_/Si interface during post-deposition anneal^38,39^, which, nevertheless, does not dilute the lifetime or iron concentration difference between b-Si and planar samples. Lastly, the role of electrically inactive phosphorus on gettering remains ambiguous^40,41^. The used diffusion process is known to result in a significant amount of inactive phosphorus near the wafer surface, which is further pronounced in b-Si^14^. The simulations confirmed an increasing amount of inactive dopants in b-Si with decreasing LTA temperature, which could partly contribute to the gettering efficiency enhancement.

## Conclusions

In conclusion, we have shown that the large surface area of b-Si, a considerable barrier for high efficiency b-Si solar cells in the past, can be used as an advantage to enhance gettering of detrimental metal impurities, specifically iron. We demonstrated that the effective minority carrier lifetime increased from less than 2 μs of an intentionally contaminated wafer to more than 1.5 ms via b-Si gettering, implying a significant increase in the substrate efficiency potential for b-Si solar cells^27^. The significant lifetime improvement was achieved by the reduction of the interstitial iron concentration from 1.7 × 10^13^ cm^−3^ to less than 10^10^ cm^−3^. A systematic series of different low temperature anneals, supported by simulations, suggested that the main gettering mechanism was segregation in both b-Si and planar samples, implying that the enhanced gettering efficiency can readily be described by predictive silicon solar cell process models^23,24^. Additionally, damage caused by b-Si fabrication could promote iron precipitation at the surface. These results hence indicate that the implementation of b-Si could enable the usage of lower quality silicon in e.g. photovoltaic devices via enhanced gettering.

### Data availability

The datasets generated and analysed during the current study are available in the figshare repository, https://figshare.com/s/709964545df554612b29.
